# Arena3D: visualization of biological networks in 3D

**DOI:** 10.1186/1752-0509-2-104

**Published:** 2008-11-28

**Authors:** Georgios A Pavlopoulos, Seán I O'Donoghue, Venkata P Satagopam, Theodoros G Soldatos, Evangelos Pafilis, Reinhard Schneider

**Affiliations:** 1 Structural and Computational Biology Unit, EMBL, Meyerhofstrasse 1, Heidelberg, Germany

## Abstract

**Background:**

Complexity is a key problem when visualizing biological networks; as the number of entities increases, most graphical views become incomprehensible. Our goal is to enable many thousands of entities to be visualized meaningfully and with high performance.

**Results:**

We present a new visualization tool, Arena3D, which introduces a new concept of staggered layers in 3D space. Related data – such as proteins, chemicals, or pathways – can be grouped onto separate layers and arranged via layout algorithms, such as Fruchterman-Reingold, distance geometry, and a novel hierarchical layout. Data on a layer can be clustered via k-means, affinity propagation, Markov clustering, neighbor joining, tree clustering, or UPGMA ('unweighted pair-group method with arithmetic mean'). A simple input format defines the name and URL for each node, and defines connections or similarity scores between pairs of nodes. The use of Arena3D is illustrated with datasets related to Huntington's disease.

**Conclusion:**

Arena3D is a user friendly visualization tool that is able to visualize biological or any other network in 3D space. It is free for academic use and runs on any platform. It can be downloaded or lunched directly from . Java3D library and Java 1.5 need to be pre-installed for the software to run.

## Background

One of the key challenges in the biosciences is finding hidden or buried information in biological networks. One of the main approaches to this challenge is to apply data analysis and clustering tools, and to visualize the results, usually in two-dimensional (2D) graphs. Some of the most prominent visualization tools are Cytoscape [1], Osprey [2], Medusa [3], ProViz [4], CNPlot [5]. Ondex [6], MAPMAN [7], Pajek [8] and Shark [9], BioLayout Express^3D ^[10].

Several of these tools provide easy connection to, or import from, commonly used external data analysis and clustering tools, such as Mega [11,12] or Hierarchical Clustering Explorer [13]. Furthermore, some visualization tools, such as Pajek [8], have analysis methods built in. We believe that tight integration of analysis and visualization is a key feature for such tools, since it allows users to experiment, to immediately see the results of analysis, and hence to quickly decide which analyses are most appropriate.

A major difficulty for these visualization tools is dealing with increasing complexity as the number of entities increases. After a certain number of nodes and edges – several hundreds or thousands – the graph becomes incomprehensible. As the biological databases grow, it is becoming increasingly apparent that the complexity of most biological process and systems simply overwhelms most graph visualization methods.

One possible solution to this problem is to visualize networks in three dimensions (3D) – a graph that is convoluted in 2D can sometimes be simplified when the same data is represented in 3D, primarily because graph vertices are much less likely to intersect in 3D. However, there are some disadvantages of 3D visualization – firstly, it can be computationally demanding; secondly, navigation in 3D requires a more complex user interface that can be difficult to learn.

In this paper, we introduce a new layer concept that can take advantage of the strength of both 2D and 3D representation; this concept has been implemented in a new visualization system, Arena3D. Arena3D allows users to take a large dataset and divide it into a series of simpler 2D graphs, each of which can be easier to comprehend, compared with all data on one 2D graph, or all data in 3D space. Separating these layers in 3D allows more 'space' for the vertices between layers, hence avoiding the overlap and intersection that occurs in 2D, and allowing visualization of a larger number of points.

In addition, Arena3D goes further than most visualization tools in integrating many different kinds of analysis methods directly with visualization. Our goal with Arena3D is to make it easier to explore and discover hidden relationships in larger, more complex data sets.

## Methods

### Implementation and requirements

The core graphics visualization in Arena3D was done using Java 3D (1.5.1 API). All other parts of Arena3D, including clustering methods and the graphical user interface, were done using Java (JDK 1.6).

To provide fast 3D interaction and navigation, we recommend using a graphics card with hardware-accelerated 3D graphics and at least 256 MB of graphical memory. Additionally, we recommend at least 1GB of RAM memory for medium to large networks, especially when using clustering methods. Prior to installing Arena3D, the user should first install the Java Runtime Environment  and Java3D . For Macintosh computers, JOGL  should also be installed. The software is freely available for non-commercial use at . Users can download the software or run it directly from the web page using java web start. Also available at this site are the Huntingtin-related datasets presented in this work.

### 3D graphics and graphical user interface GUI

Arena3D is highly interactive and incorporates many features that can be applied either to the whole network representation, to specific layers, or to individual nodes. We used the SimpleUniverse class to create a Java3D universe that implements standard GUI operations, e.g., default mouse operations, and 3D navigation such as zooming in/out, rotation, and translation.

By default, SimpleUniverse operations apply to the whole Java3D scene – in addition, we used the Java3D vecmath library to implement transformations (scaling, rotation, and translation) for individual objects, such as nodes, labels, and layers. Since these calculations are done by the graphical processing unit, these operations are fast. Arena3D allows the user to change the order, location, and orientation of individual layers. Furthermore the layer size can be adjusted and nodes can be moved anywhere on the plane of the layer they belong to. The user can save to file the entire state of the network, including all variables (coordinates, transformations, layer length and reference points). Previous states can then be restored by reloading these files.

To enable users to hide layers, nodes, labels, or connections, we used the Java3D concept of branch groups and switches. Switches were used to control whether the branch groups below that switch are visible or not. The branch groups also have the attributes to delete or create individual scene objects below them on the fly.

We implemented a jText component that allows the user to select nodes by name or using regular expressions. In addition, we enabled users to select nodes graphically by making the scene canvas selectable so that some mouse events can pick the geometry of the objects.

Furthermore we implemented some additional functionality that can make networks easier to understand. For example, we implemented a connection filter that enables users to first select a group of nodes, and then extend the selection to all nodes connected to the first selection. In addition, by simply clicking on a node or connection, the user is shown statistics and further information about that object. Finally, each node or edge can be linked to a user-defined URL (uniform resource location). For nodes with no assigned URL, the Google TouchGraph browser can be automatically launched to try to recognize the node based on its name.

### Data representation

In Arena3D, we initially arrange data in multilayered graphs in 3D, where the data are separated into different data types, one type per layer. Each layer can be moved or rotated freely in 3D space. For each node, the user can specify a connection to any other node. This connection may represent a similarity measure, such as sequence similarity for genes and proteins; it could represent experimental measurements (like Pearson correlation coefficient for micro-array expression data) or Tanimoto similarity [14] for chemicals. The connection weight could also indicate the reliability of a connection [15]. Arena3D currently supports undirected weighted graphs, where the thickness of the lines can be adjusted according to the connection weight or similarity measure. Currently Arena3D can accept only one type of connection between two nodes. In the future we are planning to support multi-edge connections.

### Layout algorithms

We implemented several simple layout algorithms to distribute the nodes on each layer; for example, nodes can be distributed randomly, on a 2D grid, spherically, or on a circle. These simple layouts do not depend on connections or similarity measures between entities, and hence can be applied to all data points on a layer.

Furthermore, we implemented two force-directed methods. The first is Fruchterman-Reingold [16], which defines the coordinates of individual objects in such a way that it minimizes the crossovers between the connections of nodes that belong to the same layer, and also it can minimize the overlaps between these nodes. The second force-directed method is based on distance geometry [17], which is appropriate when the complete distance matrix of similarities is known between all entities in one layer, unlike Fruchterman-Reingold, which only requires a subset of the distance matrix to be known. Fruchterman-Reingold is best applied to data where connectivity between nodes is relatively spare and indicates an important relationship between the entities; in contrast, distance geometry is more applicable in situations where connectivity data is generated automatically from an all-against-all comparison, e.g. from sequence or Tanimoto similarity scores. Thus, Fruchterman-Reingold tends to place the hubs (highly connected nodes) in the middle of the layer, whereas in distance geometry, all points are connected, and the layout tends to places together nodes that are connected by close similarity scores.

**Figure 1:**
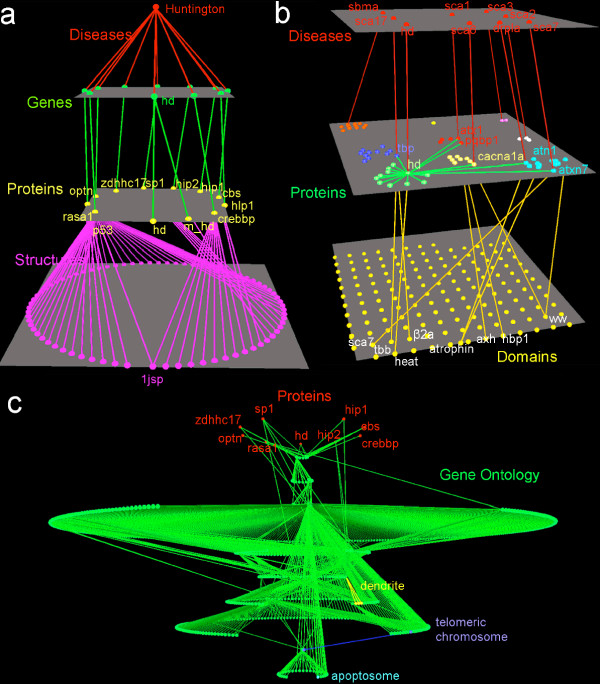
**Screenshots of Arena3D showing data related to Huntington's disease**. 1a shows the result of a query starting from Huntington's disease. HD is related to nine associated genes which are linked to 10 proteins, the Huntingtin gene 'htt' shows two forms, mutant and wild-type. These proteins link to 75 protein structures. 1b shows nine polyQ-related diseases (top layer). On the middle layer, 66 proteins known to be associated to these diseases were clustered, and on the bottom layer 151 domains associated with these 66 proteins are shown. On the middle layer we have highlighted 6 proteins that are involved in both Huntington and another polyQ disease, and on the bottom layer we have highlighted the 8 domains present in these six proteins. WW and atrophin domains are connected with proteins related to different diseases. 1c shows the proteins related to Huntingtin (top, red) and their connection to the GO ontology hierarchy.

**Figure 2:**
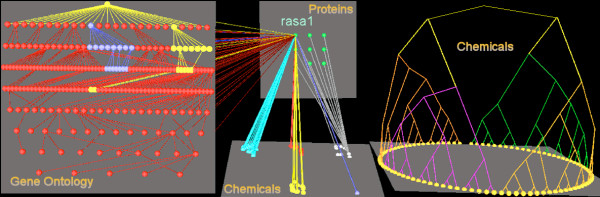
**Illustration of how Arena3D can show connections between different data types and cluster data**. The layer in the top centre position shows the 9 proteins associated with Huntingtin. We selected rasa1 protein and highlighted its connections to GO terms using our novel hierarchical layout (left image). In addition, we show connections from the nine Huntingtin-related proteins to a group of associated chemicals (bottom, center). These chemicals are shown clustered by affinity propagation, and by tree clustering (right image). In both cases the clustering is based on Tanimoto scores. The figure also illustrates how layers can be moved and rotated to allow better views on the data.

In addition we developed a third novel layout algorithm that distributes the nodes in a hierarchy depending on the connections. The algorithm assigns weights to the nodes in such a way that it ranks them and separates them in different levels forming a tree-like structure. The algorithm is fast and the resulting layout is aesthetic and informative, since it minimizes the number of crossovers. It is also suitable for visualizing any kind of hierarchical or tree-like data such as gene ontology. For example, a user can use Arena3D to visualize Gene Ontology [18] clusters produced by the POSOC [19] software. An example of hierarchical layout is shown in Figure 1c and Figure 2 (below)

### Clustering algorithms

In Arena3D, we make a distinction between layout and clustering algorithms – clustering algorithms are layout methods that organize data into a distinct number of clusters. In Arena3D we implemented the following clustering algorithms: k-means [20], affinity propagation algorithm [21] and Markov clustering [22].

In the case of the k-means, the number of clusters needs to be defined by the user, whereas the other two algorithms define the number of clusters automatically based on the data. After the data has been clustered, each node will be unambiguously coloured and identified as belonging to a one of the clusters. Affinity propagation and Markov clustering do not need the whole similarity matrix; hence they are especially applicable to sparse graphs. For dataset up to several thousand connections, clustering is usually fast (several minutes or less) – however for larger sets, clustering may require considerable time or memory. Users should be aware that, in the current implementation, affinity propagation is very stable, but is significantly slower and requires more memory than k-means or Markov clustering. Markov clustering is fast, but tends to split the data into many clusters. K-means is usually the fastest, but is very sensitive to the number of clusters that the user specifies.

### Tree clustering algorithms

In addition to the above clustering methods, we implemented the following tree-based clustering algorithms: single linkage hierarchical clustering – HCL [23,24], neighbour joining-NJ [25], and unweighted pair group method with arithmetic mean – UPGMA [26]. In each case, the user needs to provide an all-against-all similarity matrix in the input file to hold every pair-wise similarity between the nodes.

In Arena3D we implemented a method to draw tree hierarchies on each layer. We also implemented a method that allows users to graphically adjust the threshold value that determines the final number of clusters that are used to paint the leaf nodes in the tree. Different tree-based clustering methods can be applied to different layers (see Figure 2 below).

### Clustering between layers

To simplify the appearance of the whole network, we developed a method to minimize the number of intersections between inter-layer connections. Our current approach is to apply the clustering method (either Markov clustering, Affinity propagation, or k-means) to the whole network. Effectively, we cluster all points onto a single layer, and then separate the layers after the clustering. Nodes that occur in the same cluster, but which are on different layers will then occur on top of the other and will have the same colour scheme.

In any case we used a novel algorithm based on CubicBezierSegments to produce a set of contradictory colours between each other to paint the clusters

### Finding indirect connections

Very often in biological networks, important information is hidden or not easy to detect. To overcome this problem we developed some extra functionality to extract indirect connections using triplets. Arena3D can suggest new relationships by revealing indirect connections between pairs of nodes that are indirectly connected through a third intermediate node. The main idea is to extract hidden direct and indirect biological relationships, identify heterogeneous relationships, then to modify their appearance so the user can see immediately which data are selected. E.g., suppose that node A is directly connected with node B and node B is directly connected with node C, then there is an indirect connection between nodes A and C. For example, the indirect connection between fish oil and Raynaud's syndrome has been extracted via the intermediate nodes of a network that contained chemicals, phenotypes and diseases [27]. The indirect connection was found via common terms extracted with the help of text mining techniques. The link was made by finding common terms, such as blood viscosity. Indirect connections detected by Arena3D can be saved in a text file as a list of connections.

### Layer mapping

Another feature in Arena3D is the ability to map from one knowledge layer to all the others. For example, the user can select a group of nodes on one layer, and Arena3D can automatically highlight all nodes on the other layers that are connected to the selected nodes. If a node is connected to more than one of the selected nodes, it is highlighted differently; this effectively shows the 'expression profile' of each selected node, and allows the user to more easily detect overlaps and intersections between the different data sets.

### Compatibility with other tools

We developed Arena3D to work together with several other visualization tools; the entire network or graph can be exported to Medusa [3] for 3D representations, Pajek [8] for further mathematical analysis, or VRML format. Data that have been clustered using the tree-based algorithms in Arena3D can be exported to New Hampshire Format [28]. This makes Arena3D compatible with a wide variety of tools for tree visualization like for example Itol [29].

### Bioinformatics data mining

In the Results section (below) we used several sample datasets that were constructed using the following sources: SRS [30] was used for mapping connections between different databases, the String database [31] for finding protein-protein interactions, OMIM for disease information, PDB for structure information [32], GO for the Gene Ontology information [18].

## Results

Arena3D is a highly interactive, 3D visualization system – to illustrate the value of such a system, it is clearly not optimal to use only static 2D images. In this section, we present several relatively small datasets that illustrate how Arena3D can be used to gain insight into biological networks; however we refer interested readers to , where they can download the program together with several example datasets.

In Figure 1a we show how Arena3D can be used to illustrate the results of a query across multiple databases. In this case, we start the query from Huntington's disease, and we show the nine most strongly associated genes listed in the String database. These genes are then shown linked to 10 proteins on the next layer, where we indicate how one gene (Huntingtin) has two variants – mutant and wild type Huntingtin. On the next layer, we have shown all known 3D structures (75) that are linked to these proteins. One of these proteins, p53, is linked to over half of these structures, indicating that a lot of 3D information is available for this protein. In contrast, other proteins, including Huntingtin, are not associated with any known 3D structure. The graph shows one 'hidden' feature that was of interest and was not known to us before creating this view: two of the proteins associated with Huntingtin – p53 and CREB-BP – occur in the same PDB file. This means that the 3D structure of the complex of these two proteins is known.

In Figure 1b, we show a slightly more complex network – this time starting from nine polyQ-related diseases, including Huntingtin. Each of these diseases is associated with a polyQ protein, and from the String database we have found 66 proteins associated with these proteins.

On the second layer we use affinity propagation to cluster these proteins. The graph reveals several interesting and hidden features, namely that six proteins associated with Huntingtin are also associated with other polyQ disease proteins: they are: TBP (Sca17), Atn1 (DRPLA), Atxn1 (Sca1), Atxn7 (Sca7), CACNA1A (Sca6), and PQBP1 (Sca1).

On the final layer we have mapped all 151 domains present in the 66 proteins, and we have highlighted eight domains that are present in these six proteins shared with Huntingtin and other polyQ diseases. This highlighting reveals another interesting hidden feature: two domains are common in two diseases: WW is present in both ITCH (Sca7) and PQBP1 (Sca1), and the Atrophin domain is present in both Atn1 (DRPLA), and RERE (Sca7).

In Figure 1c, we show the nine proteins associated with Huntingtin mapped onto the sub-cellular location hierarchy in GO. The view is generated by finding all GO location terms in the corresponding Uniprot [33] files, finding the related GO hierarchy using POSOC [19], and using the hierarchical and circular layout methods in Arena3D. As an example for how a user would navigate through such a complex view, we have coloured terms in the GO hierarchy that are directly related to the protein rasa1 – from this we see that this protein is located in protein complexes, in the apoptosome, the telomeric regions of the chromosome, and in dendrites.

Finally, Figure 2 shows a 'full blown' illustration of how a user could work with Arena3D in practice to explore complex, large datasets. The scenario illustrated shows again the nine proteins associated with Huntingtin. In this case the rasa1 protein is selected, and its GO terms are shown together with the GO hierarchy, this time using a planar layout. For illustration, we have shown the proteins mapped onto a group of 80 related chemicals clustered via affinity propagation using Tanimoto similarity. The chemicals are also shown clustered using a tree method. In this case, to simplify the graph, connections between the tree layer and others layers have been hidden by Arena3D.

## Conclusion

In this article we presented a powerful new tool for comprehensive visualization and exploration of large scale, complex networks. Arena3D combines a novel 3D layer concept, with many layout and clustering algorithms, novel data filtering methods, flexible colour schemes, and compatibility with many existing tools. We demonstrated the functionality of Arena3D using data sets related to Huntington disease due to simplicity reasons. However Arena3D is able to handle large scale networks with hundreds of nodes with thousands of connections.

## Discussion

Future work will focus on improving usability and improving the integration with data sources and web services. We also plan to support standard formats, such as SMBL [34] or PSI-MI [35]. In addition, we plan several extensions, such as allowing multi-edge connections, directed edges, and further clustering method that minimize overlap between edges from different layers. Once directed edges can be visualized, Arena3D could then be used to represent signal transduction networks, e.g. separating different sub-cellular compartments into different layers. The strength of a tool greatly depends on the layout algorithms it can employ to make pattern easily detectable. Extension of existing layout algorithms in 3D and enrichment with new ones will be a future step.

## Authors' contributions

GAP was the main developer of the tool. SIO'D wrote the draft of the article and suggested HD related data to demonstrate the functionality of the tool. VPS as an SRS administrator helped with the collection of the data for the publication. TGS was involved in the development of affinity propagation clustering algorithm in java. EP helped with the implementation of the GUI. RS had the initial idea of the multi-layered concept of Arena3D and he was the main supervisor of this project.
